# Gonadotropin-releasing hormone agonist use in men without a cancer registry diagnosis of prostate cancer

**DOI:** 10.1186/1472-6963-8-146

**Published:** 2008-07-14

**Authors:** Yong-fang Kuo, James S Goodwin, Vahakn B Shahinian

**Affiliations:** 1 Department of Internal Medicine, University of Texas Medical Branch, Galveston, TX, USA; 2 Sealy Center on Aging, University of Texas Medical Branch, Galveston, TX, USA; 3 Department of Preventive Medicine and Community Health, University of Texas Medical Branch, Galveston, TX, USA; 4 Department of Internal Medicine, University of Michigan, Ann Arbor, MI, USA

## Abstract

**Background:**

Use of gonadotropin-releasing hormone (GnRH) agonists has become popular for virtually all stages of prostate cancer. We hypothesized that some men receive these agents after only a limited work-up for their cancer. Such cases may be missed by tumor registries, leading to underestimates of the total extent of GnRH agonist use.

**Methods:**

We used linked Surveillance, Epidemiology and End-Results (SEER)-Medicare data from 1993 through 2001 to identify GnRH agonist use in men with either a diagnosis of prostate cancer registered in SEER, or with a diagnosis of prostate cancer based only on Medicare claims (from the 5% control sample of Medicare beneficiaries residing in SEER areas without a registered diagnosis of cancer). The proportion of incident GnRH agonist users without a registry diagnosis of prostate cancer was calculated. Factors associated with lack of a registry diagnosis were examined in multivariable analyses.

**Results:**

Of incident GnRH agonist users, 8.9% had no diagnosis of prostate cancer registered in SEER. In a multivariable logistic regression model, lack of a registry diagnosis of prostate cancer in GnRH agonist users was significantly more likely with increasing comorbidity, whereas it was less likely in men who had undergone either inpatient admission or procedures such as radical prostatectomy, prostate biopsy, or transurethral resection of the prostate.

**Conclusion:**

Reliance solely on tumor registry data may underestimate the rate of GnRH agonist use in men with prostate cancer.

## Background

Prostate cancer is the most frequently diagnosed non-skin malignancy and is the third leading cause of cancer mortality in men in the United States [1]. Use of androgen deprivation therapy for prostate cancer in the form of gonadotropin-releasing hormone (GnRH) agonists is now common, with nearly half of men receiving it at some point in their disease course [2]. Historically the use of androgen deprivation therapy was limited to palliation of metastatic prostate cancer. However, the 1990s witnessed a dramatic increase in use of GnRH agonists for prostate cancer across all stages and grades [3,4], even though a survival benefit has only been demonstrated in combination with radiation in the subset of patients with locally advanced or high risk disease or following radical prostatectomy in men with node-positive cancer [5-7].

Coupled with its sizeable financial costs, the documented adverse effects of GnRH agonist therapy – including osteoporosis, fractures, sexual dysfunction, reduced quality of life and cardiovascular disease [8-12] – would argue for conservative rather than widespread use, particularly in settings where clear evidence of benefit is lacking.

Part of the popularity of GnRH agonist agents may be their ease of administration, since they can be given as once a month to every 3 or 4 month depot injections as an outpatient. We believe that these agents are sometimes prescribed without a complete evaluation for prostate cancer, based on our clinical experience of encountering patients treated on the basis of elevated prostate specific antigen (PSA) levels alone. Such cases may be missed by tumor registries, which mainly identify their cases from inpatient evaluations, surgical procedures or histopathological reports [13]. Previous population based assessments from tumor registry data in the Surveillance, Epidemiology and End Results (SEER) program may therefore underestimate the total extent of GnRH agonist use [4].

A recent study demonstrated the advantage of supplementing tumor registry data with Medicare claims for cancer ascertainment [14]. We therefore used Medicare claims from the linked SEER-Medicare database to identify men receiving GnRH agonists who had prostate cancer on the basis of claims data alone, but who were not registered as having a diagnosis of prostate cancer in SEER. We then examined the factors associated with lack of a SEER diagnosis of prostate cancer in men receiving GnRH agonists. We hypothesized that older age, comorbidity and black race would be associated with GnRH agonist use in the setting of no prostate cancer diagnosis in SEER, because previous studies have shown those characteristics to be associated with incomplete diagnostic evaluation for cancer [14-16].

## Methods

### Data Sources

This study used data from the linked SEER-Medicare database. The SEER program consists of a group of population-based tumor registries in selected geographic areas, from 11 states, covering approximately 14% of the United States population [17,18]. Medicare is a federal program that covers health services for 97% of persons aged 65 years and older. It provides data in the form of claims submitted by providers for reimbursement, which include information on diagnoses and the service, testing or procedure carried out. Data from the two programs have been linked to yield a database containing information on persons aged 65 and older with incident cancers. In addition to containing information on patients with incident cancers identified through the SEER program, the linked database also includes as a "non-cancer control" group a 5% random sample of Medicare eligible patients residing in the SEER areas who do not have a registered diagnosis of cancer by one of the SEER tumor registries. The version of the SEER-Medicare database used for this study contains Medicare claims through 2001 and SEER cancer cases through 1999.

### Study Subjects

The analysis was limited to men who received GnRH agonists for incident prostate cancer. Since we hypothesized that the factors affecting whether patients were registered in a SEER tumor registry pertained to the initial diagnostic evaluation of the cancer, only men receiving GnRH agonists within a year of diagnosis were included in the analyses.

The "SEER group" included men aged 67 years and older with incident prostate cancer registered in a SEER tumor registry from 1993 to 1999 who received at least one dose of a GnRH agonist within a year of cancer diagnosis (restricting the analysis to men receiving at least six doses did not substantially alter the results). The analysis was limited to men 67 years and older to allow assessment of Medicare claims data for at least two years prior to the diagnosis of cancer. To ensure completeness of Medicare claims data, men diagnosed by autopsy, death certificate, or not covered under both Medicare Part A and B, or were in an HMO any time from 2 years before to 1 year after their cancer diagnosis were excluded.

The "non-SEER group" included men in the 5% control group (who therefore did not have a diagnosis of prostate cancer registered in a SEER tumor registry) who had a first primary diagnosis of prostate cancer from Medicare inpatient, outpatient or physician claims from 1993 through 1999 (claims were searched from 1991) and received at least one dose of a GnRH agonist following the diagnosis (509 men were initially identified, after exclusion of 16 GnRH agonist users who did not have a claims diagnosis of prostate cancer). To ensure that the GnRH agonist use was for incident prostate cancer, men who had a claims diagnosis for a history of prostate cancer, who had their first dose of GnRH agonist more than one year after the date of the first claim diagnosis of prostate cancer, or who had any claims for GnRH agonist use prior to the first claims diagnosis of prostate cancer, were excluded (238 cases). Furthermore, as for the SEER group, the analysis was limited to men aged 67 years and older, and men not covered under both Medicare Part A and B, or who were in an HMO any time from 2 years before to 1 year after their cancer diagnosis were excluded (71 cases). Since our hypothesis was that these patients were not registered in SEER due to a less complete diagnostic work-up, we wanted to exclude patients with prostate cancer that may have failed to be registered in SEER purely due to methodological issues relating to reporting to SEER or with the SEER to Medicare linkage process. These issues include the possibility that: 1) men in the non-SEER group may have not been living in a SEER area at the time of their prostate cancer diagnosis, 2) men in the non-SEER group may have received their prostate cancer care entirely outside of a SEER area, despite being resident in a SEER area at the time of their diagnosis 3) there was a delay in reporting of the diagnosis to SEER, 4) the linkage process between Medicare and SEER files failed in some cases. Men living outside of a SEER area at the time of their prostate cancer diagnosis were identified from county code information available from Medicare, and excluded (77 cases). Men whose only hospital visits (either from inpatient or outpatient Medicare claims) for prostate cancer within a year of diagnosis occurred at a hospital located outside of their SEER state of residence were excluded (10 cases). The probabilities of a delay in reporting of the diagnosis to SEER and an imperfect linkage of SEER to Medicare data were modeled, and used to exclude men for each calendar year (21 and 7 cases, respectively). Details for these models are provided in the Statistical Methods section, below.

### Study Variables

A diagnosis of prostate cancer was identified directly from the SEER files for men who were registered in a SEER tumor registry. For the non-SEER group, a prostate cancer diagnosis was based on the presence of the relevant International classification of diseases, 9^th ^revision, (ICD-9) code (Table 1) in the first position in any inpatient, outpatient or physician Medicare claim. For both SEER and non-SEER groups, Medicare claims for the relevant ICD-9 procedure and Current Procedural Terminology, 4^th ^edition (CPT-4) codes were used to identify related procedures including radical prostatectomy, radiation treatment, transurethral resection of the prostate (TURP), pelvic computed tomographic (CT) scanning or magnetic resonance imaging (MRI) and bone scans (Table 1). GnRH agonist administration was identified through specific Medicare claims codes used to designate each dose given of certain specific injectable medications, as described previously [4].

**Table 1:** Claims definition of study variables

**Study Variable**^a^	**Claims Definition**
Prostate cancer diagnosis	ICD-9 diagnosis code 185.xx in first position
History of prostate cancer	ICD-9 diagnosis code V10.46
Radical Prostatectomy	Any of:ICD-9 procedure code 60.5 or CPT-4 codes 55801,55810,55812,55815,55821,55831,55842,55845
Radiation Treatment	Any of: ICD-9 procedure code 92.2× or ICD-9 diagnosis codes V58.0, V66.1, V67.1 or CPT-4 codes 77621–77499, 77600–77620, 77750–77799, 79200–79999 or Revenue codes 330, 333, 339, 342
Prostate Biopsy	Any of: ICD-9 procedure codes 60.1 or CPT codes 55700–55705
Transurethral resection of prostate	Any of: ICD-9 procedure code 60.2 or CPT-4 codes 52612, 52614, 52647, 52468
Transrectal ultrasound	Any of: CPT-4 codes 76872, 76873
Pelvic computed tomographic scanning or magnetic resonance imaging	Any of: ICD-9 procedure codes 88.01, 88.95 or CPT-4 codes 72192–72194, 74150, 74160, 74170–74175, 72195–72197, Health Care Common Procedure Code A9507
Bone Scan	Any of: ICD-9 procedure code 92.14, CPT-4 codes 78300, 78305, 78306, 78315, 78320

ICD-9, International Classification of Diseases, 9^th ^revision; CPT-4, Current Procedural Terminology, 4^th ^edition

^a^A diagnosis or procedure was deemed to be present if it was listed at least once in any of the outpatient, inpatient or provider Medicare claims files

Patient demographic characteristics were derived from the SEER (for region of residence at time of diagnosis) and Medicare records in the linked database. The socio-economic characteristics of each patient were based on percent of adults with less than 12 years of education from census tract data. Comorbidity was assessed using Klabunde's modification of the Charlson comorbidity index, based on information from Medicare inpatient, outpatient and physician claims in the 12 months prior to cancer diagnosis [19].

### Statistical Methods

#### Probabilities for reporting delay and imperfect SEER to Medicare linkage

We developed a model to estimate the probability that an individual patient in the non-SEER group had no prostate cancer diagnosis in SEER due to a delay in reporting of the diagnosis. Using two sets of public use SEER data reported with a lag time of three years apart, for patients diagnosed with prostate cancer from 1973–1996 (available in April 1999 [20]) and an updated dataset available in April 2002 [21], we built a multiple logistic regression model to predict the likelihood of a three year delay. For patients not listed in the earlier dataset, delay was defined as the presence in the later dataset of a diagnosis of cancer in 1996 or earlier. Predictor variables included age, race, SEER region, reporting source (based on presence or absence of an inpatient admission relating to prostate cancer) and histologic confirmation of diagnosis (based on presence or absence of a surgical procedure on the prostate). To account for the probability of delay, *p*_yr_, for periods different from three years, the results were further adjusted using data from a previous study listing the rate of incomplete records for prostate cancer diagnosis in SEER as a function of different lengths of reporting lag time [22]. The overall probability of delay, *p*_i_, for each individual patient, on the basis of patient characterstics, *βX*, was therefore calculated using the formula listed:

*p*_i _= EXP(LOG (*p*_yr_/(1-*p*_yr_)) + *βX*)/(1 + EXP(LOG (*p*_yr_/(1-*p*_yr_)) + *βX*)).

We estimated the probability of an individual patient lacking a diagnosis in SEER due to an imperfect linkage between Medicare and SEER by applying known SEER to Medicare match rates for prostate cancer (available from the National Cancer Institute) stratified by age, race and SEER region. In this adjustment process, we assumed no interactions between age, race and SEER region on the match rate.

By applying the individual probabilities of a delay, and an imperfect SEER to Medicare linkage, each patient was assigned a weighted value from 0 to 1 (with a patient receiving 0 if there was a 100% probability that they were not registered in SEER due to a delay or imperfect linkage, and a patient receiving 1 having a zero probability). Subtracting the sum of the weighted values from the total number of patients yielded the number of patients excluded due to the possibility of a reporting delay or an imperfect linkage.

#### Analyses

The number of men in the 5% control group identified as having prostate cancer by Medicare claims and receiving a GnRH agonist within one year of diagnosis (i.e., the non-SEER group) was inflated by a factor of 20. The proportion of incident GnRH agonist users that were in the non-SEER group was then calculated (with bootstrap methods used to estimate 95% confidence intervals) and stratified by year of diagnosis, age, race, SEER region, census tract education, and comorbidity. Chi-square testing was used to compare proportions across categories within each stratum. The proportion of incident GnRH agonist users who underwent hospitalization, diagnostic work-up (prostate biopsy, transrectal ultrasound), surgical procedures (TURP, radical prostatectomy), radiation treatment, or staging (CT scan or MRI, bone scan) within 3 months before or after diagnosis (date based on first Medicare claim for prostate cancer) was compared between men in the SEER and non-SEER groups using the chi-square test. Multivariable logistic regression was performed to further assess the factors associated with lack of a SEER diagnosis of prostate cancer among incident GnRH agonist users. In the regression models, lack of a SEER diagnosis of prostate cancer was treated as the dependent variable and potentially relevant study variables described above were entered as independent variables. Odds ratios (ORs) with 95% confidence intervals (CI) were calculated for each covariate. All tests of statistical significance were two-sided, with p < 0.05 being considered significant. All analyses were performed using SAS version 9.1 (SAS Institute, Cary, NC). The study protocol was approved by the Institutional Review Board of the University of Texas Medical Branch at Galveston.

## Results

A total of 17,424 men identified with incident prostate cancer in the SEER tumor registries from 1993 through 1999 received a GnRH agonist within a year of diagnosis. During the same period, a total of 85 men were identified from the 5% "non-cancer control" sample as having a Medicare claims diagnosis of incident prostate cancer and receiving a GnRH agonist within a year of diagnosis after excluding men that may not have been registered in SEER due to methodologic issues. Inflating this number by a factor of 20 to account for the 5% sampling yielded an estimate of 1700 men with prostate cancer diagnosed by Medicare claims but not registered in the SEER program who received GnRH agonists within a year of diagnosis.

Table 2 presents patient characteristics of the incident GnRH agonist users, divided as SEER group versus non-SEER group. Overall, 8.9% (95%CI: 7.8%–10.1%) did not have a prostate cancer diagnosis registered in the SEER program. A higher proportion of men 75 years and older, black, and with comorbidities had no prostate cancer diagnosis registered in the SEER program, but differences in proportions were statistically significant only for comorbidity.

**Table 2:** Patient characteristics of incident GnRH agonist users

Incident GnRH agonist users					
Characteristics	Strata	SEER group^a^	Non-SEER group^b^	Proportion not in SEER group	
		n	n^c^	%	p-value^d^
Total		17424	1700	8.9	
					
Year of diagnosis	1993	2008	190	8.6	0.26
	1994	1983	269	11.9	
	1995	2034	178	8.0	
	1996	2330	265	10.2	
	1997	2750	215	7.3	
	1998	2964	260	8.1	
	1999	3355	323	8.8	
					
Age at diagnosis (years)	67 – 74	8209	653	7.4	0.11
	≥75	9215	1047	10.2	
					
Race	White	14484	1395	8.8	0.70
	Black	1626	199	10.9	
	Other/Unknown	1314	106	7.5	
					
Region	Northeast	2570	265	9.3	0.15
	Midwest	5597	351	5.9	
	South	1331	156	10.5	
	West	7926	928	10.5	
					
Zip education	< 12.5%	4223	294	6.5	0.65
(% adult <12 years education)	≥12.5%	12090	966	7.4	
					
Comorbidity Index	0	12604	1045	7.7	0.03
	≥1	4820	655	12.0	

^a^Diagnosis of prostate cancer registered in SEER

^b^Diagnosis of prostate cancer from Medicare claims, but not in SEER, and excluding men who may have had a delay in reporting, moved out of the SEER area at the time of diagnosis or failed the SEER to Medicare linkage

^c^Sample size inflated by a factor of 20 to account for 5% sample and rounded to nearest integer

^d^Chi-square test for comparison of proportions across strata for each characteristic, based on uninflated sample sizes. P-value reported for year of diagnosis is based on test for linear trend

GnRH; Gonadotropin-releasing hormone

SEER; Surveillance, Epidemiology and End-Results

Table 3 shows the proportion of incident GnRH agonist users with various diagnoses and procedures within 3 months before or after diagnosis of prostate cancer, comparing those with a diagnosis of prostate cancer from a SEER tumor registry versus those with a diagnosis based only on Medicare claims. Men in the non-SEER group were less likely to undergo either inpatient admission or a procedure that would lead to diagnosis or histologic confirmation of prostate cancer (TURP, prostate biopsy, transrectal ultrasound or radical prostatectomy), with 87.0% in the SEER group versus 70.8% in the non-SEER group undergoing at least one of the above (p < 0.01 for difference in proportions). Men in the non-SEER group had lower rates of staging work-up (bone scan or CT scan/MRI) but the differences did not achieve statistical significance. They were also significantly more likely to be treated with GnRH agonists without concurrent radiation treatment.

**Table 3:** Diagnoses and procedures in incident GnRH agonist users, comparing those with and without a diagnosis of prostate cancer in the SEER tumor registries

Diagnoses and procedures in incident GnRH agonist usersa					
	SEER group^b^		Non-SEER group^c^		
	n	%	n^d^	%	p-value^e^
**Procedures related to diagnosis and histologic confirmation of prostate cancer**^a^					
Hospitalization	3691	21.2	338	19.9	0.75
Transurethral resection of prostate	1504	8.7	56	3.3	0.08
Prostate Biopsy	13728	79.0	1153	67.8	0.01
Transrectal Ultrasound	10342	59.5	868	51.1	0.11
Radical Prostatectomy	766	4.4	51	3.0	0.47
Any of the above	15122	87.0	1204	70.8	<0.01
					
**Other treatment/work-up**^a^					
Radiation Therapy	3156	18.2	85	5.0	<0.01
CT scan/MRI pelvis	6421	36.9	503	29.6	0.16
Bone Scan	11048	63.6	910	53.5	0.06

^a^Within 3 months before or after diagnosis of prostate cancer (date based on Medicare claims)

^b^Diagnosis of prostate cancer registered in SEER

^c^Diagnosis of prostate cancer from Medicare claims, but not in SEER and excluding men who may have had a delay in reporting, moved out of the SEER area at the time of diagnosis or failed the SEER to Medicare linkage

^d^Sample sizes inflated by a factor of 20 to account for 5% sample and rounded to nearest integer

^e^Chi-square test for comparison of proportions between SEER and non-SEER group, based on uninflated sample sizes

GnRH; Gonadotropin-releasing hormone

SEER; Surveillance, Epidemiology and End-Results

Table 4 presents the results of two multivariable logistic regression models examining factors predicting lack of a prostate cancer diagnosis in a SEER tumor registry among incident GnRH agonist users. Model 1 includes patient characteristics from Table 2 entered as independent variables. Model 2 additionally includes diagnostic procedures from Table 3 entered as independent variables. In the first model, greater comorbidity significantly increased the likelihood that an incident GnRH agonist user did not have a diagnosis of prostate cancer listed in the SEER program. There were also significant, two-fold variations by SEER geographic region. In the second model, receipt of TURP or use of radiation treatment were associated with a lower likelihood that an incident GnRH agonist user lacked a diagnosis of prostate cancer in the SEER program. We also performed a third model (not shown) entering the composite variable from Table 3 of receipt of at least one of: hospitalization, TURP, transrectal ultrasound, radical prostatectomy or prostate biopsy. This was associated with a significantly reduced likelihood that an incident GnRH agonist user was not registered in the SEER program (OR 0.38; 95%C.I. 0.22–0.65).

**Table 4:** Predictors of lack of a diagnosis of prostate cancer in the SEER tumor registries among incident GnRH agonist users

			Model 1^a^		Model 2^b^	
Variable	Category	Distribution (%)	Odds Ratio	95% CI	Odds Ratio	95% CI
Year of diagnosis	1993	11.5	1.00		1.00	
	1994	11.8	1.35	(0.59,3.10)	1.34	(0.58,3.09)
	1995	11.6	0.88	(0.35,2.19)	0.88	(0.35,2.20)
	1996	13.6	1.13	(0.49,2.61)	1.14	(0.49,2.65)
	1997	15.5	0.78	(0.33,1.88)	0.81	(0.34,1.95)
	1998	16.9	0.85	(0.37,1.98)	0.89	(0.38,2.07)
	1999	19.2	0.91	(0.41,2.04)	0.96	(0.43,2.17)
						
Age at diagnosis (years)	67 – 74	46.3	1.00		1.00	
	≥75	53.7	1.38	(0.89,2.15)	1.24	(0.79,1.95)
						
Ethnicity	White	83.0	1.00		1.00	
	Black	9.6	1.30	(0.66,2.58)	1.23	(0.62,2.46)
	Other	7.4	0.67	(0.27,1.65)	0.68	(0.28,1.68)
						
Region	West	46.3	1.00		1.00	
	Northeast	14.8	0.86	(0.46,1.60)	0.84	(0.45,1.57)
	Midwest	31.1	0.51	(0.29,0.91)	0.46	(0.26,0.83)
	South	7.8	0.78	(0.36,1.71)	0.79	(0.36,1.72)
						
Comorbidity Index	0	71.4	1.00		1.00	
	≥1	28.6	1.60	(1.03,2.48)	1.55	(1.00,2.41)
						
Zip education	< 12.5%	25.7	1.00		1.00	
(% adult < 12 years education)	≥12.5%	74.3	1.24	(0.68,2.28)	1.24	(0.68,2.26)
						
**Procedures within 3 months before or after diagnosis**						
Hospitalization		21.2			1.48	(0.79,2.79)
Transurethral resection of prostate		8.4			0.19	(0.05,0.68)
Prostate Biopsy		77.9			0.59	(0.33,1.06)
Transrectal Ultrasound		58.7			0.96	(0.57,1.62)
Radical Prostatectomy		4.5			0.51	(0.13,2.00)
Radiation Therapy		17.1			0.27	(0.10,0.72)
CT scan/MRI pelvis		36.3			1.08	(0.63,1.85)
Bone scan		62.6			0.86	(0.52,1.44)

^a^Multivariable logistic regression model with presence or absence of a diagnosis of prostate cancer in SEER as the dependent variable, and year of diagnosis, patient age, race, SEER region of residence, comorbidity index, and educational status entered as independent variables

^b^Multivariable logistic regression model with presence or absence of a diagnosis of prostate cancer in SEER as the dependent variable, and the variables from Model 1 with the addition of the listed diagnostic procedures entered as independent variables

GnRH; Gonadotropin-releasing hormone

SEER; Surveillance, Epidemiology and End-Results

## Discussion

The results of our study can be summarized as follows. Reliance solely on cases of prostate cancer registered in the SEER program appears to underestimate incident GnRH agonist use by about 9%, even after accounting for methodologic issues that may have affected registration or reporting. Among incident GnRH agonist users, lack of a diagnosis of prostate cancer in the SEER program was more likely in men with comorbidities whereas it was less likely for men who had undergone procedures such as TURP, prostate biopsy, or radiation treatment.

Based on the results of this study, what can be inferred about GnRH agonist users without a tumor registry diagnosis of prostate cancer? They tend to be older, of black race, and have more comorbidities, all typical of patient characteristics associated with less complete diagnostic evaluations for cancer [14,15]. GnRH agonist users without a diagnosis in SEER had lower rates of procedures which allow histologic confirmation of the diagnosis, an important element for cancer registration, as compared to those registered in SEER (Table 3) [15]. Nevertheless, two thirds of men in the non-SEER group underwent prostate biopsy within 3 months of their claims diagnosis of prostate cancer. This may suggest that even with an available histologic diagnosis, registration in SEER is further facilitated when there is an inpatient admission, or surgical procedure such as radical prostatectomy, which were both somewhat more common in the SEER group. Alternatively, the results of the prostate biopsies noted in the non-SEER group may have either been negative or equivocal. Follow-up PSA testing is common following a negative prostate biopsy, and a rising level may have potentially led to treatment with GnRH agonists in the absence of a definitive histologic diagnosis [23].

Given the nature of cancer registration, prostate cancer cases managed with surgery or radiation are most likely to be captured due to the frequent hospital contacts (either through inpatient admission or visits to hospital outpatient departments). In contrast, men treated with GnRH agonists alone may frequently be managed entirely with physician office visits. The finding that a proportion of GnRH agonist users with prostate cancer are missed by the SEER registries is therefore not surprising. Nevertheless, if the proportion of GnRH agonist users without a SEER registry diagnosis is extrapolated to national figures (with 40% of the over 200,000 new prostate cancer cases in the United States receiving GnRH agonists), it could translate to several thousand men a year [1,4]. It is especially important to be able to monitor use of GnRH agonists in men with prostate cancer given limited evidence of efficacy, particularly in men with substantial comorbidity, and the increasing recognition of life threatening adverse effects such as bone fracture and cardiovascular disease [11,12,24-27].

There are limitations to our study. First, we made an assumption that the distribution of subject characteristics in the 5% control sample could be extrapolated to the entire non-cancer population, inflating the sample size by a factor of 20. This assumption might not hold true, particularly with extensive stratification. We therefore minimized the number of strata within each characteristic. Second, the sample size was small, limiting the statistical power to draw inferences about factors affecting registration in SEER among GnRH agonist users. Third, in order to ensure that demographic information was comparable between the SEER and non-SEER groups, we were limited to Medicare as the source for both. Medicare claims do not allow for direct assessment of marital status, a potentially important predictor of cancer work-up [28], and are of limited utility in identifying race/ethnicity categories other than White or Black [29]. In addition, there may be error introduced in the assessment of some of the variables using Medicare claims. However, we used previously validated methods for important variables such as comorbidity and GnRH agonist use [19,30]. Furthermore, since we used similar methods of assessment for both the SEER and non-SEER groups, any misclassification as a result of using claims data would tend to bias the results towards the null, such that the significant associations noted in Table 4 would still be valid. Fourth, despite the multiple methodologic exclusions we imposed, the 8.9% figure for incident GnRH agonist users that are not captured by the SEER registries is likely to be an overestimate. For example, in the non-SEER group, some cases classified as incident GnRH agonist use may in fact have been prevalent use, due to the difficulty in defining date of cancer diagnosis with Medicare claims alone. To estimate the impact of this, we applied the Medicare claims algorithm to SEER GnRH agonist users (who have an accurate date of diagnosis provided by the registry), finding that 90.4% were correctly classified as incident use. In addition, we were only able to exclude men with hospital-based prostate cancer care provided outside of the SEER state of residence, since information on location was only available for hospitals. Finally, only subjects age 67 or older with Medicare Part A and B enrollment but without HMO membership were studied, so the results may not necessarily apply to younger men or patients with other forms of health insurance.

## Conclusion

Reliance solely on data from tumor registries may underestimate the rate of GnRH agonist use in men with prostate cancer.

## Competing interests

The authors declare that they have no competing interests.

## Authors' contributions

YK participated in the conception and design of the study, the drafting of the manuscript, and performed all the statistical analyses. JSG participated in the conception and design of the study, and the drafting of the manuscript. VBS participated in the conception and design of the study, and the drafting of the manuscript. All authors read and approved the final manuscript.

## Pre-publication history

The pre-publication history for this paper can be accessed here:


